# Editorial: Cell signaling in cancer metastasis and lineage plasticity

**DOI:** 10.3389/fcell.2023.1302659

**Published:** 2023-10-13

**Authors:** Wei Yang, Kavita Shah, Rong Yang

**Affiliations:** ^1^ Department of Pathology and Cancer Center, Stony Brook University, Stony Brook, NY, United States; ^2^ Department of Chemistry, Purdue University Center for Cancer Research, Purdue University, West Lafayette, IN, United States; ^3^ Department of Urology, Affiliated Drum Tower Hospital, Medical School of Nanjing University, Institute of Urology, Nanjing University, Nanjing, China

**Keywords:** signaling, metastasis, lineage plasticity, ONECUT2, PAK1, network, LncRNA―long non-coding RNA

Metastasis, the primary cause of cancer deaths, remains a poorly understood malignant process (Gerstberger et al., 2023). While the last two decades have witnessed the emergence of numerous targeted therapies for metastatic cancers, their effectiveness often diminishes quickly due to the development of drug resistance. Among the drug resistance mechanisms, lineage plasticity, which is the ability of cells to change from one committed developmental pathway to another (Quintanal-Villalonga et al., 2020), is gaining recognition as an important factor. However, the molecular mechanisms underlying tumor cell plasticity remains largely uncharted. The objective of this Research Topic is to delve into recent advancements in understanding the molecular underpinnings of cancer metastasis or lineage plasticity, with the ultimate goal of enhancing our comprehension and management of these life-threatening processes.

Recent research has unveiled One Cut homeobox 2 (ONECUT2) as a pivotal regulator of cancer metastasis and lineage plasticity (Rotinen et al., 2018; Guo et al., 2019; Liu et al., 2021). Intriguingly, Steadman et al. have revealed that ONECUT2 mRNA functions as a master competing endogenous RNA (ceRNA) with its extraordinarily lengthy 3′ untranslated region (UTR) spanning 14,575 nucleotides. Even more fascinating is the discovery that both the ONECUT2 3’ UTR and the ONECUT2 protein activate similar gene networks to promote cancer aggressiveness, indicating a cooperative functional relationship between the two ONECUT2 entities.

The p21-activated kinase-1 (PAK1) is a key player in various biological processes and a critical factor in cancer progression and metastasis (Yao et al., 2020). Saldivar-Ceron et al. have uncovered a novel mechanism by which PAK1 promotes breast tumorigenesis. Their study demonstrates that PAK1/CaMKII signaling is required for the efficient proliferation, migration, and invasion of mammary epithelial cells, suggesting that co-targeting PAK1 and CaMKII holds promise as a therapeutic approach for breast cancer.

In a Hypothesis and Theory article, Harryman et al. propose that the heterogeneity of cell-cell and cell-extracellular matrix adhesion receptors drives aggressive tumor networks and has significant functional implications, particularly in muscle-invasive and lethal carcinomas. They hypothesize the existence of functional epithelial-mesenchymal cooperation (EMC) within early tumor invasive networks, facilitating tumor escape from the primary organ and navigation to metastatic sites. Understanding EMC in the early tumor invasive networks could lead to the identification of early biomarkers for aggressive transitions and the discovery of novel agents to inhibit the EMC phenotype.

H19, the first long non-coding RNA discovered (Brannan et al., 1990), has been found to play a key role in various diseases including cancer. In a comprehensive review article, Wu et al. summarized H19’s key roles in cancer development and progression, as well as its regulatory functions in oncogenic signaling pathways such as the PI3K/Akt, canonical Wnt/β-catenin, canonical NF-κB, MAPK, JAK/STAT, and apoptotic pathways. The accumulating evidence suggests that H19 holds promise as both a therapeutic target and a biomarker for cancer diagnosis, prognosis, and treatment.

In summary, this Research Topic offers insights into the ongoing research advancements in cell signaling related to cancer metastasis and lineage plasticity. These efforts are expected to enhance our understanding of the fundamental mechanisms governing these processes, ultimately paving the way for more effective therapeutic strategies to combat these life-threatening conditions.
